# AB-QTL analysis reveals new alleles associated to proline accumulation and leaf wilting under drought stress conditions in barley (*Hordeum vulgare* L.)

**DOI:** 10.1186/1471-2156-13-61

**Published:** 2012-07-20

**Authors:** Mohammed A Sayed, Henrik Schumann, Klaus Pillen, Ali A Naz, Jens Léon

**Affiliations:** 1 University of Bonn, Institute of Crop Science and Resource Conservation, Crop Genetics and Biotechnology Unit, Katzenburgweg 5, Bonn, 53115, Germany; 2 Martin-Luther-University Halle-Wittenberg, Institute for Agricultural and Nutritional Sciences, Plant Breeding, Betty-Heimann-Str. 3, Halle, 06120, Germany

**Keywords:** QTL analysis, Drought stress tolerance, Proline content, Leaf wilting

## Abstract

**Background:**

Land plants have evolved several measures to maintain their life against abiotic stresses. The accumulation of proline is the most generalized response of plants under drought, heat or salt stress conditions. It is known as an osmoprotectant which also acts as an instant source of energy during drought recovery process. But, both its role and genetic inheritance are poorly understood in agriculture crops. In the present work, advanced backcross quantitative trait locus (AB-QTL) analysis was performed to elucidate genetic mechanisms controlling proline accumulation and leaf wilting in barley under drought stress conditions.

**Results:**

The analysis revealed eight QTL associated to proline content (PC) and leaf wilting (WS). QTL for PC were localized on chromosome 3H, 4H, 5H and 6H. The strongest QTL effect *QPC.S42.5H* was detected on chromosome 5H where drought inducible exotic allele was associated to increase PC by 54%. QTL effects *QPC.S42.3H*, *QPC.S42.4H* and *QPC.S42.6H* were responsible to heighten PC due to the preeminence of elite alleles over the exotic alleles which ranged from 26% to 43%. For WS, QTL have been localized on chromosome 1H, 2H, 3H and 4H. Among these, *QWS.S42.1H* and *QWS.S42.4H* were associated to decrease in WS due to the introgression of exotic alleles. In addition, two digenic epistatic interaction effects were detected for WS where the additive effect of exotic alleles imparted a favorable increase in the trait value.

**Conclusions:**

The present data represents a first report on whole-genome mapping of proline accumulation and leaf wilting in barley. The detected QTL are linked to new alleles from both cultivated and wild accessions which bring out an initial insight on the genetic inheritance of PC and WS. These QTL alleles are fixed in the isogenic background of Scarlett, which will allow for positional cloning of underlying genes and to develop drought resilient barley cultivars.

## Background

Water is fundamental to life and its shortage can cause unprecedented risks to the survival of flora and fauna. Land plants suffer more because of their sessile mode and hence, drought is by far the most devastating abiotic stress that limits agricultural production worldwide [1-3]. In this scenario, the development of drought-adaptive cultivars is essential to reduce crop losses in agriculture.

Barley (*Hordeum vulgare* L.) is the fourth important cereal crop and considered as a genomic model for the tribe *Triticeae.* It is characterized by immense variation with respect to drought adaptation and has been cultivated from boreal to equatorial regions of the world [4]. In this regard, the wild progenitor of barley, *H. vulgare* ssp. *spontaneum,* has a real potential of maintaining a stable population in the desert conditions of Middle East. Genetic dissection of such novel adaptation is a prime question towards understanding drought stress tolerance in plants. The slow progress in this area is mainly due to quantitative inheritance of drought related traits. The advent of molecular tools has made it possible to dissect genetic inheritance of this trait-complex and several successful QTL analyses have been performed in barley and related species [5-16]. These studies revealed that crop plants evolved a number of drought adaptive traits to maintain their life in water deficit conditions. For instance, leaf rolling is an interesting adaptation to conserve internal water by reducing the transpirational losses under drought stress conditions [5]. Inability of this process may result in leaf wilting and death of leaves because of failure to cope with the transpiration demands of plants [17]. Price et al. [18] reported eleven QTL for leaf drying which contributed significantly to drought avoidance of upland rice. In sorghum, Sanchez et al. [7] identified four major QTL for stay-green, the most important agronomic trait for sorghum cultivation under drought conditions. These data showed that leaf wilting (drying) is a vital drought adaptive trait which offers a straightforward determination of drought tolerance in plants. Therefore, it has been used in large scale screenings of drought tolerance under field conditions [19]. No report of QTL mapping for leaf wilting was found in barley.

The knowledge created in model species suggested that plants inherit cues of physiological responses which determine or regulate the development of different drought adaptive traits. Among these, accumulation of free proline is a major and the most studied cellular signal of plants under stress conditions. It is primarily synthesized from glutamate via two successive reductions catalyzed by Δl pyrroline-5-carboxylate synthetase (*P5CS*) and *P5C* reductase (*P5CR*), respectively [20]. It acts as a widely distributed osmosolute that protects plants against drought, salinity or low temperature etc. [21,22]. Its higher reserves can be converted back to glutamate which in turn becomes a prompt source of energy during drought recovery period [23,24]. It has also been reported that the accumulation of proline products and catabolism of glutamate can result in the expression of several drought inducible genes in rice [25]. Further, its significance has also been reported under control conditions in fulfilling diverse functions during plant development like rapid cell division, floral transition and embryo development [24,26-29]. These reports highlight a diverse role of proline metabolism in plant development under both control and stress conditions in model plants but little is known about its significance in the process of drought stress tolerance in agricultural crops. For instance, its levels were tested to screen drought stress tolerance in barley where higher proline level was found in drought susceptible genotypes [30,31], which has created a significant confusion about its modulation in the mechanism of drought stress tolerance in crop species. One major reason behind this lacking is the poor understanding of its genetic inheritance in crops.

The present study was focused on the dissection of the genetic inheritance of proline content (PC) and the associated trait leaf wilting (WS) in barley. We report the first whole-genome QTL map for PC and WS that reveals the identification of associated QTL alleles from both cultivated and exotic origins. Furthermore, the role of stable and drought inducible QTL alleles as well as the digenic epistatic interactions in the determination of QTL effects associated to PC and WS under drought stress conditions has been investigated.

## Results

### Variance analysis of PC and WS

A variance analysis of proline content (PC) and wilting score (WS) among the population S42, across treatments and years, is presented in Table 1. It reveals significant variation for PC among BC_2_DH lines of the population S42, between treatments, S42 by treatment interaction, across years and block by (treatment x year). For WS, highly significant variation were found among population S42, between treatments, across years and block by (treatment x year) except for the S42 by treatment interaction effect. A lower heritability of PC was observed among the 301 BC_2_DH lines but these lines displayed heritable responses for WS at h^2^ =0.75.

**Table 1:** Mutli-factorial analysis of variance of PC and WS among population S42

**Trait**^**1**^	**SOV**^**2**^	**DF**^**3**^	**F-value**^**4**^	**P-value**^**5**^	**(h**^**2**^**)**^**6**^
PC	S42	300	1.18	<0.05	0.15
	Treatment	1	850.41	<0.001	-
	S42 x Treatment	300	1.27	<0.01	-
	Year	2	539.89	<0.001	-
	Block (Treatment x Year)	32	16.23	<0.001	-
WS	S42	300	3.99	<0.001	0.75
	Treatment	1	2415.57	<0.001	-
	S42 x Treatment	300	0.96	ns	-
	Year	2	30.34	<0.001	-
	Block (Treatment x Year)	32	15.52	<0.001	-

^1)^Traits; PC (proline content) and WS (leaf wilting score), ^2)^source of variance, ^3)^degree of freedom, ^4)^F-value, ^5)^P-value (<0.05, <0.01, <0.001), ^6)^heritability of traits among BC_2_DH population S42.

### Phenotypic characterization of PC and WS

**Figure 1:**
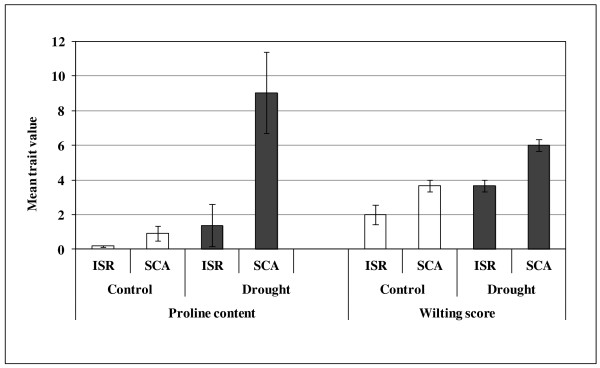
**Comparison of PC (μmol/gDW) and WS (from 0 to 9) between ISR42-8 (ISR) and Scarlett (SCA) under control and drought stress conditions.** Trait values have been averaged across years 2007, 2008 and 2009. PC was measured according to Bates et al. [32]. WS was assessed by a visual scoring from 0 to 9 according to de Datta et al. [33].

The parents, Scarlett and ISR42-8 showed significant variation for PC and WS under drought stress and control conditions (Figure 1). Scarlett revealed a remarkable increase of PC from 0.8 μmol/gDW (control) to 4.7 μmol/gDW under drought stress whereas ISR42-8 showed a modest increase in PC in drought block as compared to control. On average, Scarlett accumulated around 3.3 μmol/gDW more PC than ISR42-8 under drought stress conditions. Likewise, Scarlett showed a mean WS of 2.5 under control conditions that increase up to 5.0 under drought stress condition whereas ISR42-8 presented a slight increase in WS from 2.0 (control block) to 3.7 (drought stress block).

**Figure 2:**
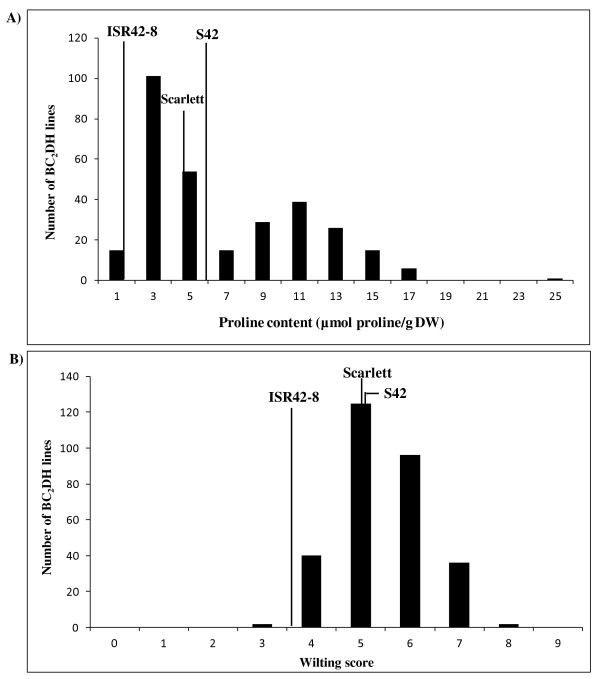
**Frequency distribution of population S42 for PC (A) and WS (B) under drought stress condition.** The vertical lines represent mean trait value of Scarlett, ISR42-8 and population S42. PC was measured according to Bates et al. [32]. WS was assessed by a visual scoring from 0 to 9 according to de Datta et al. [33].

The population S42 showed a range of PC values with a mean of 5.9 μmol/gDW under drought stress conditions (Figure 2A). A total of 15 BC_2_DH lines accumulated the least amount of PC (1.0 μmol/gDW) under drought stress. The largest group of 101 BC_2_DH lines produced 3.0 μmol/gDW of PC. The second largest group contained 54 BC_2_DH lines which synthesized around 5 μmol/gDW of PC. Altogether, 131 BC_2_DH lines showed values of PC that exceeded Scarlett and the mean value of population S42. In these genotypes, the PC ranged from 7.0 to 25.0 μmol/gDW. For WS, population S42 showed a significant variation and presented a mean WS of 5.1 under drought stress treatment (Figure 2B). Two BC_2_DH lines showed the lowest (resistant) WS at 3.0 while around 40 BC_2_DH lines displayed WS 4.0. A total of 125 and 96 BC_2_DH lines showed leaf wilting severity at 5.0 and 6.0 respectively. Around 38 BC_2_DH lines appeared to be highly susceptible under drought and accounted for WS 7.0 and 8.0.

To dissect the relationship among PC and WS, Pearson correlation coefficient (*r*) were calculated which indicated a significant but weak correlation between PC and WS traits (*r* = 0.2, P < 0.001).

### QTL analysis

The phenotypic and genotypic data has been subjected to QTL analysis for the identification of QTL associated to PC and WS (Table 2).

**Table 2:** Summary of QTL for PC and WS

**QTL**^**1)**^	**Chr**^**2)**^	**Marker**^**3)**^	**Range**^**4)**^	**Effect**^**5)**^	**F-value**^**6)**^	**P-FDR**2^**7)**^	***R***^**2 8)**^** (%)**	**[*Hsp*]**^**9)**^	**RP[*Hsp*]**^**10)**^** (%)**	***Add***^**11)**^
Proline content										
*QPC.S42.3H*	3H	bPb-4628	175.2	M, M x T	23.6	< 0.01	6.1	2.1	- 43	- 0.77
*QPC.S42.4H*	4H	EBmac635	130-138	M, M x T	15.5	< 0.05	4.2	2.5	- 27	- 0.47
*QPC.S42.5H*	5H	MGB338	95	M x T	14.0	< 0.01	4.1	4.8	54	0.89
*QPC.S42.6H*	6H	Bmag613	68-84.6	M, M x T	20.5	< 0.01	4.0	2.6	- 26	- 0.44
Wilting score										
*QWS.S42.1H*	1H	HvABAIP	94.9 - 123.9	M	43.4	< 0.01	12.0	3.4	- 17	- 0.27
*QWS.S42.2H*	2H	bPb-4261	38.9 - 44.7	M	28.4	< 0.01	5.6	4.7	22	0.43
*QWS.S42.3H*	3H	bPb-9110	118.7 - 141.9	M	211.4	< 0.01	34.0	5.0	35	0.48
*QWS.S42.4H*	4H	VrnH2	140.2 - 146	M	21.5	< 0.01	9.4	3.4	- 17	- 0.18

^1)^Description of quantitative trait locus. ^2)^Chromosome. ^3)^Linked DNA marker revealing strongest F-value ^4)^CentiMorgan positions of associated DNA markers from the first to the last significant marker in a QTL region. ^5)^Effects showing by the QTL, marker main (M) and marker by treatment (M*T) interaction effects. ^6)^F-value of the given marker locus. ^7)^Probability of false discovery rate at P < 0.05. ^8)^Genetic variance explained by M or M x T. For calculation see von Korff et al. [34]. ^9)^Trait value of homozygous exotic genotypes *Hsp* in terms of Lsmeans across years.^10)^Relative performance of the homozygous exotic allele, RP*Hsp*, a “-“indicates a reduction in trait value. ^11)^The additive effect is half the difference between the phenotypic means of the homozygous elite and exotic marker genotypes. Traits; PC (proline content) and WS (leaf wilting score).

### QTL for PC

**Figure 3:**
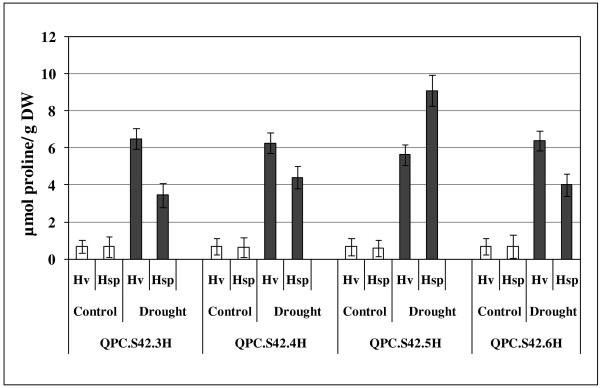
**Marker by treatment interaction effect for PC.** Lsmeans of elite (Hv) and exotic (Hsp) alleles for PC under control and drought conditions were compared. Vertical lines indicate standard error of the mean.

The QTL analysis revealed four QTL for PC located on chromosomes 3H, 4H, 5H and 6H. The strongest QTL effect, *QPC.S42.5H* was detected on chromosome 5H where an exotic allele accounted for a 54% increase in PC and asserted the highest positive additive effect (0.89). Linked marker to this QTL showed a marker by treatment (M x T) interaction effect and explained 4.1% of the explained genetic variance. The remaining three QTL, *QPC.S42.3H*, *QPC.S42.4H* and *QPC.S42.6H* showed a decreasing trend of PC due to the introgression of exotic alleles. These QTL effects revealed a preponderance of elite alleles over the exotic alleles for PC. At QTL, *QPC.S42.3H* the elite allele showed 43% increase in PC with respect to the exotic allele and explained 6.1% of the explained genetic variance. Similarly, the relative performance of exotic alleles at QTL, *QPC.S42.4H* and *QPC.S42.6H* was 26% and 27% less than the respective elite alleles. Here, each QTL represented around 4% of the genetic variance (Table 2, Figure 3).

### QTL for WS

Reduced WS under drought is a desirable trait and therefore, QTL effects which accounted for lower WS, represent favorable leads for drought tolerance. QTL analysis revealed four QTL for WS on chromosomes 1H, 2H, 3H and 4H. Among these, two favorable exotic alleles at QTL, *QWS.S42.1H* and *QWS.S42.4H* were associated to almost 17% decrease in WS. These exotic alleles explained 12.0% and 9.4% of the explained genetic variance, respectively. In contrast, the exotic alleles at QTL, *QWS.S42.2H* and *QWS.S42.3H* were associated with an enhancement of WS as compared to elite alleles. Here, elite alleles appeared to contribute in decreasing WS of which the elite allele at QTL, *QWS.S42.2H* posed a 22% superior response as compared to the exotic allele and accounted for 5.6% of the explained genetic variance. Likewise, the relative performance of the elite allele at *QWS.S42.3H* was 35% higher than the respective exotic allele (Table 2).

### Epistatic interaction effects

**Figure 4:**
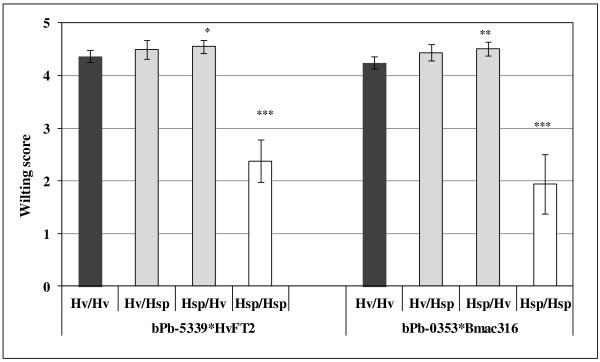
**Digenic epistatic interaction effects for WS.** Lsmeans of four genotypes, Hv/Hv (elite allele at loci 1 and 2), Hv/Hsp (elite allele at locus 1 and exotic allele at locus 2), Hsp/Hv (exotic allele at locus 1 and elite allele at locus 2), Hsp/Hsp (exotic allele at loci 1 and 2). Genotype combinations with significantly different Lsmeans when compared with the control genotype Hv/Hv are marked with asterisks (***P < 0.001, **P < 0.01, *P < 0.05). Vertical lines indicate standard error of the mean.

Digenic epistatic interactions have been tested for PC and WS among 301 BC_2_DH genotypes. No epistatic interaction effect was found for PC whereas two interaction effects were detected for WS (Figure 4). The first interaction effect was identified between marker locus *bPb5339* (1H) and *HvFT2* (3H). At these loci, the combination of *Hv*/*Hv* or *Hv*/*Hsp* resulted in mean WS 4.4 which reduced to 2.3 as the elite allele was substituted with exotic allele at *bPb5339* (1H). In the second effect, a similar interaction of exotic alleles was detected at marker locus *bPb-0353* (3H) and *Bmac316* (6H) which dropped WS from 4.2 (*Hv*/*Hv*) to 1.9 (*Hsp*/*Hsp*).

## Discussion

The present study reports on the genetic dissection of PC and WS by using 301 BC_2_DH lines of a cross between barley cultivar Scarlett and wild accession ISR42-8. An advanced backcross population was utilized for a straightforward detection and introgression of favorable exotic alleles in the Scarlett background according to Tanksley and Nelson [35]. Our data showed a significant variation of PC and WS between parents as well as among the BC_2_DH lines. This population showed skewed frequency distributions for both traits because normal distribution is not expected in a BC_2_DH population. Mather and Jinks [36] proposed a bias to one of the parent when two parents bear difference for a trait because of successive backcrossing of the recurrent parent. The present work reports on high resolution QTL mapping data by using the biggest double haploid population in barley. It is accepted that the strength of a QTL analysis primarily depends upon the size of mapping population and the density of markers on the genetic map [37].

QTL analysis identified four QTL for PC where at three QTL the elite alleles from Scarlett were associated to heighten PC. These findings agree with higher PC in the susceptible parent Scarlett which suggests that higher proline levels may define its drought sensitivity. Proline level was used as a metabolic measure to screen drought stress tolerance in barley and higher PC were found in drought susceptible genotypes [30,31] which agrees with the response of Scarlett in the present study. It has been reported that proline levels vary considerably in different plant organs. It was found higher in reproductive organs than vegetative as well as in the organs bearing endogenously controlled dehydration, e.g. in seeds or pollen [38-40]. However, in barley leaves the preferential accumulation of proline in epidermis and vascular bundles was only observed under stress conditions [41], suggesting proline induction a reliable marker for the measurement of drought stress response in barley. Our data accounted for the preeminence of Scarlett's alleles at QTL effect *QPC.S42.3H*, *QPC.S42.4H* and *QPC.S42.6H* to enhance PC as compared to respective exotic alleles. Interestingly, these effects revealed both M and M x T interaction effects thus indicating their role under control and drought stress conditions. The knowledge gained in the model plant *Arabidopsis* suggests that proline biosynthesis occurs both in normal and stress conditions. Under normal conditions, it is synthesized to maintain the housekeeping function of the cell. Altogether, three enzyme coding genes, *P5CS1* and *P5CS2* and *P5CR* have been described to regulate proline biosynthesis in *Arabidopsis*. Under drought, the expression of *P5CS1* and *P5CR* increased which result in higher proline synthesis in the chloroplast whereas *P5CS2* is primarily linked to housekeeping proline synthesis in the cytosole [23,24]. The map position of *P5CS1* and *P5CS2* and *P5CR* are not known in barley and hence the identification of *QPC.S42.3H*, *QPC.S42.4H* and *QPC.S42.6H* on chromosomes 3H, 4H and 6H may provide an initial knowledge of proline biosynthetic loci in barley.

The strongest QTL effect was detected on chromosome 5H, where an exotic allele enhanced PC by 54%. This exotic allele showed an M x T interaction effect only which suggests that *QPC.S42.5H* may underlie a drought inducible gene of wild origin possibly similar to transcriptional activity of *P5CS1* or *P5CR* which induced under drought stress conditions [23]. However, it is still an open question whether *QPC.S42.5H* underlies an exotic variant of *P5CS1* or *P5CR* or a new component of proline biosynthesis in barley. To address this question candidate gene approach will be adapted to test DNA sequence polymorphism and expression differences of barley orthologs in Scarlett and ISR42-8. Alternatively, QTL bearing ILs are available for positional cloning of *QPC.S42.5H*. A single report was found on the genetic mapping of PC in barley where Siahsar and Narouei [42] reported two QTL for salt stress related to proline accumulation on chromosome 5H by using 72 double haploid lines of a cross between Steptoe and Morex. To see the effect of PC on plant performance we measured plant biomass which revealed major shoot weight QTL were associated to Scarlett's alleles suggesting a possible linkage drag of the exotic alleles on shoot biomass as expected from wild barley accession ISR42-8 being inferior in shoot development with respect to Scarlett (data not shown). Therefore, the effect of PC variation on biomass production may not be visible in such mixed background but candidate IL can be used to test its effect on plant performance. Although, the role of proline is still a debate in crop plants, its utility has been validated in the process of drought stress tolerance in *Arabidopsis* and rice via transgenic approaches [43-45]. Therefore, more efforts are needed to generalize these effects in crops like barley.

The extent of leaf WS is a straightforward criterion to measure a plant's ability to tolerate water limiting conditions which may be linked to drought inducible metabolites like proline. Our data revealed highly significant but weak correlation of PC and WS. Stewart [46] studied proline accumulation in wilted barley leaves and found that wilting caused 40 fold stimulation of proline biosynthesis in non-starved leaves when compared to starved leaves. He suggested the role of carbohydrates which appeared to supply precursors for proline bio-synthesis. These data indicate that although leaf wilting may stimulate proline biosynthesis, it was unable to modulate the process of its biosynthesis which agrees with the partial dependence of PC and WS. No co-localization of QTL was found which also suggest an independent genetic inheritance of both traits. Our data showed four QTL for WS where at *QWS.S42.1H* and *QWS.S42.4H* the introgression of exotic alleles was associated to reduce WS under drought stress conditions. Although these QTL are genetically independent to PC, they may still have significance in modulating proline metabolism which requires lower wilting environment. These findings support the idea of introgressing favorable exotic alleles from a tolerant wild accession ISR42-8 into Scarlett where higher PC may be ceased because of precocious leaf wilting and death under drought stress condition. Abscisic acid (ABA) is one major factor implicated for leaf drying (senescence) under severe drought [47]. Quarrie et al. [48] made a QTL analysis to dissect the genetics behind ABA accumulation and found ten QTL for low and high ABA accumulation in rice under drought stress conditions. In a previous study, they found a QTL for ABA accumulation on 5A of wheat in the region of vernalization gene *Vrn1 *[49]. Thus, the localization of *QWS.S42.4H* at marker locus *VrnH2* may indicate a putative orthologous region of *Vrn1* variant in barley. *QWS.S42.1H* was linked to HvABAIP where Tondelli *et al*. [10] described QTL for drought stress tolerance in barley. HvA1 (group 3 LEA protein), which is known as ABA induced barley effector gene, was mapped at the similar region on chromosome 1H whose overexpression confers dehydration tolerance in transgenic rice plants [50,51]. QTL effects *QWS.S42.2H* and *QWS.S42.3H* were associated to superior performance of elite alleles for decrease in WS. These findings suggest that the susceptible parent Scarlett also harbors useful alleles for WS that may underlie valuable components of shoot development and their replacement with detrimental exotic alleles might be a reason behind such variation. Strikingly, all four QTL for WS showed no M x T interaction effect suggesting significant and stable QTL effects across control and drought stress blocks. A possible reason may lie in the genetic control of WS where the associated QTL alleles are active across both treatments. Alternatively, it is possible that QTL were unable to pass the criteria of M x T interaction effects due to error created by different environmental (E) conditions across years as phenotypic evaluations were made in each year in single replication. It is worthwhile to mention that QTL x E (years) was not the focus of the present study because the experiments were carried out inside a tunnel and not under real field conditions. Furthermore, two epistatic interactions were detected where exotic alleles presented an additive role in the development lower WS. Here, the drought sensitive elite allele seems epistatic to drought tolerant exotic alleles which overshadowed the performance of exotic alleles in the determination of drought tolerance. A tolerant WS is evident in the donor parent ISR42-8 of these exotic alleles. However, lower wilting due to the interaction of a flowering gene specific marker HvFT2 on chromosome 3H is an interesting outcome which suggests a putative role of flowering as a regulator in drought stress tolerance. Wang et al. [52] identified DNA polymorphism in the 3'UTR of HvFT2 between Scarlett and ISR42-8. Von Korff et al. [34] studied heading date variation in population S42 and found at most of the ten QTL identified that the introgression of the wild allele was associated with enhanced flowering. They indicated early and heterogeneous flowering habits in wild barley were presumably linked to adaptation in a water limiting environment. These data also highlight the necessity of digenic interactions effect for a high resolution QTL analyses. The role of epistatic interactions has been accepted crucial for the determination of a final phenotype of quantitative traits [53,54].

## Conclusions

**Figure 5:**
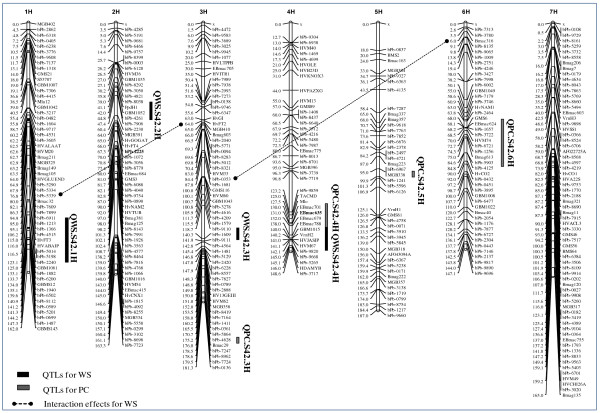
**QTL map for PC and WS in barley.** QTL for PC and WS are marked with grey and black bars, respectively. Digenic epistatic interactions have been highlighted with dotted lines where arrow heads indicate associated markers on both sides. Linkage map was drawn using MapChart ver.2.2 where the markers and genetic positions are presented on right and left of the chromosome, respectively [55].

The present study brings out a QTL map that reveals a new insight into the genetic inheritance of PC and WS in barley (Figure 5). It suggests that higher proline accumulation merely defines the extent of drought sensitivity of Scarlett. However, the significance of higher proline content in drought tolerance may be conditioned by its utility during the drought recovery period. Unfortunately, most of drought susceptible genotypes like Scarlett fail to utilize proline reserves efficiently because of early leaf senescence (wilting) and leaf death which results in proline reduction during drought conditions. To complement this lacking natural adaptation ISR42-8 seems suitable since it bears low wilting QTL alleles which may facilitate proline metabolism during drought recovery period. Therefore, these exotic alleles seem important genetic resources to decrease drought sensitivity of cultivated varieties. However, further experiments are needed in a near isogenic background to dissect a precise influence of these QTL effects on plant performance. The above mentioned leads are fixed in an isogenic background and currently introgression lines are available for further phenotyping, marker assisted selection as well as for positional cloning of underlying genes.

## Methods

### Plant material

A double haploid mapping population designated as S42 was used for QTL analysis. It consisted of 301 BC_2_DH lines which were achieved from 76 original BC_2_F_1_ lines of a cross between German spring barley cultivar Scarlett (*H*. *vulgare* L.) with the wild accession ISR42-8 (*H*. *vulgare* ssp. *spontaneum*) originating from Israel. The cultivar Scarlett was used as the recurrent parent whereas ISR42-8 was utilized as the donor of the drought related traits. The development of the BC_2_DH population was according to von Korff et al. [56]. The proportion of donor genome in this population is given in von Korff et al. [56] and Schmalenbach et al. [57].

### Genotyping of population S42

The population S42 was genotyped with simple sequence repeats (SSRs), diversity array technology (DArT) and gene-specific marker systems. A linkage map of 371 genetic markers has been established that contains 106 SSRs, 255 DArT and 10 gene-specific DNA markers. The SSR markers and gene-specific markers were according to von Korff et al. [56] and Wang et al. [52], respectively. For DArT genotyping, DNA of population S42 and both parents was isolated by using DNeasy 96 Plant Kit (Qiagen, Hilden, Germany). The DNA was sent to Diversity Arrays Technology P/L-Triticarte P/L, Yarralumla ACT, Australia for genotyping [58]. At each locus, homozygous elite or homozygous exotic genotypes have been scored as (*Hv*) and (*Hsp*), respectively. The chromosomal positions of the DArT markers are according to Wenzl et al. [59]. A linkage map has been drawn by using MapChart ver.2.2 [55].

### Experimental setup

Phenotypic characterization of the population S42 was repeated three times during the summer seasons 2007, 2008 and 2009 inside the plastic foliar tunnels at the Institute of Crop Science and Resource Conservation, Faculty of Agriculture, University of Bonn. A total of 12 seed of individual BC_2_DH line, Scarlett and ISR42-8 were sown in 22 x 22 x 26 cm plastic pots containing a mixture of top soil, silica sand, milled lava and peat dust (Terrasoil®, Cordel & Sohn, Salm, Germany). The pots were arranged in a split-plot design where individual BC_2_DH lines and parents were assigned randomly to the two irrigation blocks with the control and the drought treatments. The drought stress treatments were applied at growth stage ranging from 28 to 32 (BBCH) among BC_2_DH lines across 2007, 2008 and 2009.

Watering was done with a drip irrigation system (Netafim ™, Adelaide, Australia) by watering each pot three times a day with a calculated amount of water. Echo2 sensors (Decagon Dev., Pullman, WA, USA) were used to determine the volumetric soil moisture content (VMC) digitally with the frequency domain technique. For this purpose, before sowing eight sensors were installed in the pots of the reference cultivar Scarlett within a depth of 10 cm, four in control pots and four in the pots of the drought treatment where data were collected manually and controlled every day. Comparing these moisture data with the mass and the developmental stage and the transpiration conditions for the next three days from the weather forecast, further irrigation was determined in such a way, that in the control block the VMC remains at field capacity and in the drought treatment the VMC follows a planned curve from the VMC of field capacity (40%) down to the VMC of near wilting point (15%). The plants were fertilized three times per season with 250 ml of NPK liquid fertilizer containing 7% N, 3% P_2_O_5_ and 6% K_2_O. The plants were sprayed against fungi and insects as recommended for barley cultivation if needed. Environmental conditions across 2007, 2008 and 2009, are presented in Table 3.

**Table 3:** The average temperature and relative humidity across year 2007, 2008 and 2009 at Poppelsdorf field station Bonn, Germany

	** 2007**		** 2008**		** 2009**	
Months*	**Temp (°C)**	**RH (%)**	**Temp (°C)**	**RH (%)**	**Temp (°C)**	**RH (%)**
April	13.2	60.2	8.5	73.3	12.5	71.7
May	14.9	70.6	15.9	65.0	14.2	71.6
June	17.7	74.9	17.0	72.2	15.5	72.7
July	17.8	69.4	18.1	72.8	18.8	69.1
Average	15.9	68.7	14.8	70.8	15.3	71.2

* The values showed average temperature (°C) and relative humidity (RH) across each month in 2007, 2008 and 2009.

### Phenotypic evaluation

For the phenotypic evaluation of the population S42 proline content (PC) and wilting score (WS) were measured. For PC, a total of eight fully expanded leaves of two main tillers were harvested (four leaves each plant) separately in each control and drought stress blocks at the end of the drought period (growth stage around BBCH 41–49). The leaves were cut and wrapped in plastic foil, then frozen in liquid nitrogen, freeze-dried and ground to a fine powder by the help of a milling device (Retsch, Hann, Germany). PC was measured by colorimetric procedure according to Bates et al. [32]. For this, a total of 30 mg of the powder was homogenized in 2 ml of 3% sulphosalicylic acid by vortexing 3 times for 15 sec and centrifuged at 14000 rpm for 10 min. A total of 500 μl of the supernatant was taken in a falcon tube and increased to a volume of 1000 μl by adding 3% sulphosalicylic acid. Later, 1000 μl of ninhydrin acid and 1000 μl of glacial acetic acid were added. Then falcon tubes were vortexed for 15 sec and the resulting mixture heated to 100°C for 1 hour in a water bath. The reaction was stopped by placing tubes in an ice bath. Afterwards, 2 ml of toluene was added to each tube and vortexed for 20 sec. Subsequently, tubes were kept at room temperature for 5 min for a phase separation and the absorbance of chromophore containing toluene (blank) was read at 520 nm using a spectrophotometer. The proline concentration was determined with a standard curve method, using known concentrations of L-proline (P-0380, Sigma-Aldrich®) and calculated on a dry weight basis by the formula:

$$\begin{array}{l} Proline\;\left(\mu mol\;proline/gDW\right)\\ \;=\left(\mu g\;proline/ml\times 4\times 10\;\right)/0.03\times \;115.1 \end{array}$$

For WS, cumulative response of 12 plants of each genotype for leaf wilting (WS) was scored at the end of drought period by visual rating from 0 up to 9 where 0 represents all leaves green and 9 all leaves apparently dried [33].

### Statistical analysis

Statistical analysis was performed by using SAS software where analysis of variance (ANOVA), heritability (h^2^) and least square means (Lsmeans) were calculated using PROC MIXED procedure [60].

### QTL analysis

QTL analysis is to a great extent a model selection. Broman and Speed [61] and Bauer *et al*. [62] compared the forward selection strategy in REML analysis with Bayesian techniques and found that forward selection strategy is very effective to detect QTL associated to quantitative traits. We employed a multiple QTL model iteratively extended and reduced by forward selection and backward elimination, respectively using the PROC MIXED procedure in SAS [60]. This QTL model bears the ability to utilize individual observations of each trait value simultaneously across year, blocks and therefore, trait values were not averaged across years for marker trait analysis. For QTL analysis with fixed marker effects, it compares the marker variances and the error variance in the F-test. These effects showed a normal distribution there and hence we did not use any data transformation. According to this model, in each round of the forward selection (or backward elimination as the QTL can be excluded also) process, the most informative marker was added as a fixed factor (QTL) into the model and then all remaining markers were scanned with the respective model containing the previously found QTL. In this multiple QTL model, the following iterations were continued until no more additional QTL could be detected. Starting point was the following mixed hierarchical model:

$$\begin{array}{ll} X_{\mathrm{ijklmn}} & =\mu +M_{i}+L_{j}\left(M_{i}\right)+T_{k}+L_{j}*T+M_{i}*T_{k}\\ & \;+Y_{1}+T_{k}*Y_{l}+B_{m}\left(T_{k}*Y_{l}\right)+\varepsilon_{n\left(ijklm\right)}\text{,} \end{array}$$

where the total phenotypic value was the sum of general mean *μ*, fixed effect *M*_*i*_ of the *i*-th marker genotype, random effect *L*_*j*_*(M*_*i*_*)* of the *j*-th DH line nested in the *i*-th marker genotype, fixed effect *T*_*k*_ of the *k*-th treatment, random interaction effect *L*_j_*T_*k*_ of the *j*-th DH line and the *k*-th treatment, fixed interaction effect *M*_i_*T_*k*_ of the *i*-th marker genotype and the *k*-th treatment, fixed effect *Y*_*l*_ of the *l*-th year, fixed interaction effect *T*_k_*Y_*l*_ of the *k*-th treatment and *l*-th year, random effect *B*_*m*_*(T*_k_*Y_*l*_*)* of *m*-th block nested in treatment and years, residue ε_*n*(*ijklm*)_ of *X*_*ijklmn*_. P values from F-tests were adjusted genome-wide across all single marker tests using the probability of false discovery rate (P_FDR_), implemented in the SAS procedure PROC MULTTEST. The significant marker main effects as well as marker by treatment interaction with P_FDR_ < 0.05 were accepted as putative QTL for the next iteration. The final model was:

$$\begin{array}{ll} X_{\mathrm{ijklmn}} & =\mu +{\sum QTL+M}_{i}+L_{j}\left(M_{i}\right)+T_{k}+L_{j}*T_{k}\\ & \;+M_{i}*T_{k}+Y_{1}+T_{k}*Y_{l}+B_{m}\left(T_{k}*Y_{l}\right)\\ & \;+\varepsilon_{n\left(ijklm\right)}\text{,} \end{array}$$

where *∑QTL* represents the detected QTL from the forward/backward selection process.The contribution of a QTL to trait genotypic variance was estimated by the *R*^*2*^ coefficient (percentage of the explained genotypic variance) according to von Korff et al. [34]. Relative performance of an exotic allele RP*Hsp* to the corresponding elite allele was used as a measure of trait improvement that was calculated by the formula (RP*Hsp* = *Hsp* - *Hv*/*Hv* * 100). *Hsp* and *Hv* represent the Lsmeans of trait value of the homozygous exotic and elite genotypes, respectively.

### Digenic epistatic effects

The digenic epistatic interactions between marker pairs were tested in 301 BC2DH lines with the SAS procedure PROC MIXED using the following mixed hierarchical model:where *M1*_*i*_ and *M2*_*j*_ are the fixed effects of the *i*-th and *j*-th markers, respectively. *M1*_i_*M2_*j*_ is the fixed interaction effect of the *i*-th *M1* genotype with *j*-th *M2* genotype. *L*_*k*_*(M1*_i_*M2_*j*_*)* is the random interaction effect of *i*-th *M1* and *j*-th *M2* markers nested in *k*-th BC_2_DH line. Epistatic effects were accepted based on probability of false discovery rate (P_FDR_ < 0.05) and has been calculated by PROC MULTTEST procedure in SAS [63].

## Abbreviation

AB-QTL: Advanced backcross quantitative trait locus; PC: Proline content; WS: Wilting score; M: Marker main effect; M x T: Marker by treatment interaction effect; M x E: Marker by environment interaction effect; RP: Relative performance.

## Competing interests

The authors declare that they have no competing interests.

## Authors’ contributions

JL has conceptualized the research and MAS, HS carried out phenotypic experimentation. JL, KP and MAS contributed in achieving genotyping data. JL has written the program for QTL analysis and JL, MAS and AAN analyzed that data. JL, AAN and MAS have written the manuscript. All authors approved the final version of the manuscript.
